# Plasma cell‐free DNA markers predict occult metastases in patients with resectable pancreatic ductal adenocarcinoma

**DOI:** 10.1002/ctm2.70573

**Published:** 2026-01-19

**Authors:** Jacob E. Till, Ofer Gal‐Rosenberg, Sophia G. Giliberto, Nicholas J. Seewald, Dominique G. Ballinger, Heather E. Samberg, Melinda R. Yin, Qiao‐Li Wang, Samuele Cannas, Kristine N. Kim, Kyle Tien, Mohammed Sawi, Vidya Madineedi, C. Sloane Furniss, Vasilena Gocheva, Jonathan Nowak, Lauren K. Brais, Chen Yuan, Michael H. Rosenthal, Robert Roses, Ronald DeMatteo, Major Kenneth Lee, Charles Vollmer, Hersh Sagreiya, Mark H. O'Hara, Ruth Shemer, Brian Wolpin, Yuval Dor, Erica L. Carpenter

**Affiliations:** ^1^ Department of Medicine Division of Haematology‐Oncology, University of Pennsylvania Perelman School of Medicine Philadelphia Pennsylvania USA; ^2^ Department of Developmental Biology and Cancer Research Hebrew University Institute for Medical Research, Israel‐Canada Jerusalem Israel; ^3^ Department of Biostatistics, Epidemiology, and Informatics University of Pennsylvania Perelman School of Medicine Philadelphia Pennsylvania USA; ^4^ Department of Medical Oncology Dana‐Farber Cancer Institute Boston Massachusetts USA; ^5^ Department of Radiation Oncology University of Pennsylvania Perelman School of Medicine Philadelphia Pennsylvania USA; ^6^ Department of Pathology Brigham and Women's Hospital Boston Massachusetts USA; ^7^ Department of Radiology Harvard Medical School Boston Massachusetts USA; ^8^ Department of Surgery University of Pennsylvania Perelman School of Medicine Philadelphia Pennsylvania USA; ^9^ Department of Radiology University of Pennsylvania Perelman School of Medicine Philadelphia Pennsylvania USA

1

Dear Editor,

Detecting pancreatic ductal adenocarcinoma (PDAC) early can yield dramatic improvements in overall survival (OS). Curative intent resection is typically indicated when the disease is localised to the pancreas. However, standard of care imaging lacks sensitivity to detect smaller occult metastases, often resulting in patients undergoing an unnecessary and morbid surgery, followed by early recurrence.^1^, ^2^ While we have previously demonstrated detection of early‐stage PDAC using exocrine pancreas methylation markers in cfDNA,^3^ here we show that methylation markers, when combined with circulating tumour *KRAS* mutation detection and imaging measurements, can predict the presence of occult metastatic disease before curative intent surgery.

A convenience sample of patients was enrolled with written informed consent at the University of Pennsylvania Hospital (Philadelphia, PA), under IRB Protocol #822028, NCT02471170. Patients had previously untreated PDAC or were seen in the endoscopy clinic for routine screening (healthy controls) or non‐cancer disease evaluation and monitoring (disease controls). Disease control patients’ diagnoses included pancreatic cyst, pancreatitis, intraductal papillary mucinous neoplasm, and other non‐cancerous pancreatic conditions. Patients with PDAC were excluded for 1) insufficient imaging surveillance to identify occult metastases within 120 days of surgery or 2) receiving therapy for a second primary tumour ≤5 years of PDAC diagnosis. Clinical and demographic data were abstracted from the electronic medical record, including the presence of metastases within 120 days of surgery. Pathologic staging (pT and pN) was obtained for patients who completed surgery; otherwise, clinical staging was used. CA19‐9 values for 69 of 75 naive resectable PDAC patients were abstracted from the medical record for a timepoint within 40 days of surgery. For 6 patients, an aliquot of previously frozen plasma was provided to the clinical laboratory at the University of Pennsylvania and analysed using the clinical protocol. See Supplemental Digital Content for elaboration of study methods. This study was performed in accordance with STARD 2015 guidelines.

We analysed plasma from a cohort of 176 patients, including PDAC and non‐PDAC controls (Figure S1 and Tables S1 and S2), to explore whether cfDNA methylation markers (Figure S2 and Table S7), independent of tumour genomic profiling, distinguished PDAC patients with and without occult metastases. Building on the previous pancreas tissue methylome analysis,^3^ we identified methylated or unmethylated loci in liver and lung tissue, the two most common sites of distant metastases for PDAC. We then adapted our methods to detect these loci in plasma cfDNA. For 75 patients with PDAC who had surgery without receiving neoadjuvant therapy (“naïve resectable”), the cfDNA concentration from exocrine pancreas, hepatocytes, and lung epithelium (expressed as genome equivalents or copies per ml) was significantly higher than disease (see Methods) or healthy controls (Figure 1A). While pancreas and lung concentrations were significantly higher in 24 patients with imaging‐confirmed metastatic disease at diagnosis (“metastatic”) (Table S3) compared to naïve resectable patients, there was no significant difference in liver cfDNA (Figure 1A).

**FIGURE 1:**
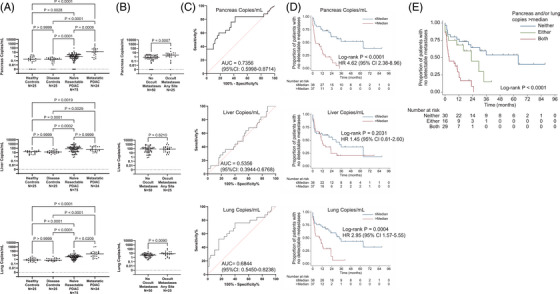
cfDNA biomarkers. (A) Methylation analysis of cell‐free DNA extracted from patient plasma was used to quantify the levels of pancreas (top), liver (centre), and lung copies/mL (bottom) for healthy controls, disease (non‐cancer) controls, naïve resectable pancreatic ductal adenocarcinoma (PDAC), and metastatic PDAC patients. *p*‐Values generated using Dunn's Multiple Comparison test. (B) Among naïve resectable PDAC patients, cfDNA biomarker levels were further analysed for those with no occult metastases versus occult metastases at any site, with associated Mann‐Whitney *p*‐values. (C) Shown is receiver‐operator characteristic (ROC) analysis for each of the three cfDNA biomarkers for the prediction of occult metastases. (D) Shown are Kaplan‐Meier analyses dichotomised on the median copies/mL for each cfDNA biomarker to predict time to first detection of metastatic disease (measured from the day prior to surgery, to allow inclusion of patients with metastases discovered intraoperatively). (E) Kaplan‐Meier curves for time to first detection of metastatic disease when both pancreas and lung copies are above the median, either is above, or neither is.

Among 75 patients with naïve resectable PDAC, 25 had occult metastases, with six discovered intraoperatively and 19 by imaging within 120 days postoperatively. Overall survival (OS) was significantly shorter for patients with versus without occult metastases (Figure S3A). Liver was the most prevalent site of first detected occult metastases (Tables S4 and S5). Pancreas and lung cfDNA were significantly higher for patients with versus without occult metastases (*p* = 0.0007 and *p* = 0.0090, respectively). However, there was no significant difference in hepatocyte copies for these two groups (*p* = 0.6210, Figure 1B). Pancreas and lung copies had significant AUCs for predicting occult versus no occult metastases (*p* = 0.0009 and *p* = 0.0096, respectively), but hepatocyte copies did not (*p* = 0.6170, Figure 1C; associated cutoffs and statistics shown in Figure S4A). Kaplan‐Meier analysis for time to any metastases (TTM) for all 75 naïve resectable patients showed that pancreas copies above versus ≤ median was associated with significantly shorter median TTM. Similar results were obtained for lung cfDNA, while no significant difference was found for hepatocyte cfDNA (Figure 1D; Kaplan‐Meier analysis using AUC‐derived cutoffs shown in Figure S4B). Patients with both pancreas and lung cfDNA above median had the shortest median TTM compared to those with either or neither marker above median (Figure 1E), with similar results for OS (Figure S3B). cfDNA methylation markers remained significantly associated with TTM and OS when patient characteristics were added in a multivariable analysis (Table S6).

We next assessed two additional blood‐based markers, pre‐surgery circulating tumour DNA‐based *KRAS* mutation detection (ctKRAS) and CA19‐9, as well as primary tumour volume as measured from pre‐surgery imaging. Among naïve resectable patients, the proportion of patients with detected ctKRAS was significantly higher for those with versus without occult metastases (*p* < 0.0001); however, there was no difference in CA19‐9 (*p* = 0.4161, Figure 2A). Primary tumour volume for naïve resectable patients with occult metastases was higher than for those without (*p* = 0.0260, Figure 2A). ROC analysis was consistent with this for CA19‐9 and tumour volume (Figure 2B), as was Kaplan‐Meier analysis for TTM and OS for ctKRAS, CA19‐9, and tumour volume (Figure 2C and Figure S5). Given that none of the continuous variables were significantly correlated with the cfDNA markers (Figure 2D), we assessed whether combining markers could improve prediction. A least absolute shrinkage and selection operator model selected pancreas and lung copies/mL, ctKRAS, and tumour volume for predicting occult metastases (Figure 2E,F).

**FIGURE 2:**
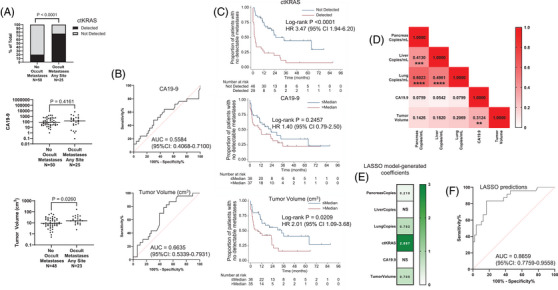
Additional non‐invasive biomarkers. (A) Among 75 naïve resectable pancreatic ductal adenocarcinoma (PDAC) patients, biomarkers were compared for patients with (*N* = 25) and without occult metastases (*N* = 50). The presence of a circulating tumour DNA‐based *KRAS* mutation (ctKRAS) was detected by ddPCR for patients with and without occult metastases, shown with Fisher's exact test *p*‐value (top). CA19‐9 (middle) and tumour volume in cm^3^ (bottom, as measured by a board‐certified radiologist (HS) from standard of care imaging of the primary pancreatic tumour) were analysed for those with no occult metastases versus metastases, shown with associated Mann‐Whitney *p*‐values. Of 75 patients with naïve resectable disease, tumour volume could only be calculated for 71. (B) Shown are receiver‐operator characteristic (ROC) analyses for CA19‐9 (top) and tumour volume (bottom) for the prediction of occult metastases. No ROC analysis was performed for the binary detected versus not detected ctKRAS assay. (C) Kaplan‐Meier analysis dichotomised on ctKRAS detected versus not detected (top), CA19‐9 (middle) and tumour volume (bottom) to predict time to first detection of metastatic disease (measured from day prior to surgery, to allow inclusion of patients with metastases discovered intraoperatively). (D) Shown are the Spearman rho values for correlative analysis of each continuous biomarker as compared to every other continuous biomarker. As a binary variable, ctKRAS was not included. Colour indicates the magnitude of the rho value, stars indicate significance (** *p* < 0.01, *** *p* < 0.001 and **** *p* < 0.0001). (E) Results of the LASSO model depicted as a heat map of the magnitude of coefficients for selected independent variables, with all analyte values normalised by log10 transformation and standardised (NS = not selected), and (F) associated ROC analysis for the prediction of occult metastases for 71 patients with complete data (both F and G).

As an exploratory analysis, we analysed cfDNA markers for 27 patients who received neoadjuvant therapy; however, the markers were not predictive of occult metastases (Figure S6).

Plasma cfDNA methylation markers may improve identification of patients with occult PDAC metastases, providing a potentially actionable biomarker for patient stratification, independent of tissue molecular analysis. These results are consistent with the recent finding that pre‐operative ctDNA levels improved disease stratification for patients with early‐stage non‐small cell lung cancer^4^ and suggest that tumours with occult metastases associate with higher rates of cellular turnover in the primary or metastatic sites. More work is needed to examine whether the concept applies to tumours beyond PDAC, and to reproduce the findings in additional patient populations to facilitate clinical implementation.

## AUTHOR CONTRIBUTIONS

E.L.C. and Y.D. conceptualised the research plan. O.G.‐R., D.B., S.C., M.Y., K.T., M.S., S.G., H.E.S., K.N.K., M.H.O., J.E.T., C.V., R.S. and H.S. generated data by experimentation or medical chart review. E.L.C., Y.D., M.Y. and R.S. managed the project. E.L.C., R.S. and N.J.S. led the primary data analysis. E.L.C. drafted the initial manuscript. S.G., R.S., E.L.C., J.E.T., O.G.‐R. and H.E.S. produced figures and tables. S.C., S.G., H.E.S. and O.G.‐R performed data organisation and conducted additional analysis. J.E.T. and E.L.C. completed the final review.

## FUNDING INFORMATION

This study was supported by the James and Marlene Scully Liquid Biopsy Innovation Fund and Penn Pancreatic Cancer Research Centre Netter Fund to Erica L. Carpenter. The Hale Family Centre for Pancreatic Cancer Research, Lustgarten Foundation Dedicated Laboratory Program, and National Cancer Institute of the National Institutes of Health Award CA210171 supported Brian Wolpin. Grants from EU (PANCAID, 101096309), the Soyka Pancreatic Cancer Fund and the Israel Innovation authority supported Yuval Dor.

## ETHICS STATEMENT

All subjects received written informed consent according to the Declaration of Helsinki, under University of Pennsylvania Institutional Review Board protocol 822028.

## Supporting information



Supporting Information

Supporting Information

Supporting Information

Supporting Information

Supporting Information

Supporting Information

Supporting Information

Supporting Information

Supporting Information

Supporting Information

Supporting Information

Supporting Information

Supporting Information

Supporting Information

## Data Availability

The data produced and analyzed for this study are included in Table S1.
