# Supplementary material for: Plasma cell‐free DNA markers predict occult metastases in patients with resectable pancreatic ductal adenocarcinoma
Source: Clin Transl Med. 2026 Jan 19;16(1):e70573. doi: 10.1002/ctm2.70573 (PMC12813551; doi:10.1002/ctm2.70573)
Supplement: Supplementary file 2 — Supporting Information [file CTM2-16-e70573-s007.pdf]

Supplemental Table 2 – Patient characteristics

	Healthy Control	Disease Control	Naïve Presurgical	Neoadjuvant Resected PDAC	Metastatic
	**25**	**25**	**75**	**27**	**24**
**Age**					
Median (Min-Max)	59 (26-80)	63 (24-85)	72 (50-85)	66 (51-81)	66 (54-81)
<65, n (%)	16 (64%)	13 (52%)	15 (20%)	12 (44%)	10 (42%)
>65, n (%)	9 (36%)	12 (48%)	60 (80%)	15 (55%)	14 (58%)
**Sex, n (%)**					
Female	11 (44%)	12 (48%)	34 (45%)	13 (48%)	15 (63%)
Male	14 (56%)	13 (52%)	41 (55%)	14 (52%)	9 (37%)
**Race, n (%)**					
African American	4 (16%)	0 (0%)	9 (12%)	5 (19%)	2 (8%)
Asian	1 (4%)	1 (4%)	3 (4%)	0 (0%)	0 (0%)
Caucasian	17 (68%)	22 (88%)	63 (84%)	20 (74%)	21 (88%)
Other (see note)	3 (12%)	2 (8%)	0 (0%)	2 (7%)	1 (4%)
**Ethnicity, n (%)**					
Hispanic or Latino	1 (4%)	0 (0%)	0 (0%)	1 (4%)	0 (0%)
Not Hispanic or Latino	24 (96%)	25 (100%)	75 (100%)	26 (96%)	24 (100%)
**Baseline ECOG Performance Status, n (%)**					
0			8 (11%)	6 (22%)	5 (21%)
1			9 (12%)	17 (63%)	15 (63%)
2			2 (3%)	0 (0%)	1 (4%)
n/a	n/a	n/a	56 (75%)	4 (15%)	3 (13%)
**CA19-9 (U/mL)**					
Median (Min-Max)	n/a	n/a	97 (<0.1-6597)	109 (12-438)	1556 (7-889390)

"Other" race includes the following: unknown, mixed race, and race not listed.
