# Supplementary material for: Plasma cell‐free DNA markers predict occult metastases in patients with resectable pancreatic ductal adenocarcinoma
Source: Clin Transl Med. 2026 Jan 19;16(1):e70573. doi: 10.1002/ctm2.70573 (PMC12813551; doi:10.1002/ctm2.70573)
Supplement: Supplementary file 3 — Supporting Information [file CTM2-16-e70573-s008.pdf]

Supplemental Table 3 – Sites of metastases as detected at time of diagnosis by imaging for metastatic cohort (N=24)

ID	Sites of metastases at diagnosis
UPENN 053	Liver
UPENN 058	Liver
UPENN 074	Portacaval, retroperitoneal, and abdominal lymph nodes
UPENN 069	Liver and retroperitoneal Lymph Nodes
UPENN 067	Liver
UPENN 064	Lung, Peritoneum
UPENN 059	Liver
UPENN 062	Peritoneum
UPENN 072	Liver, Peritoneum
UPENN 070	Liver
UPENN 073	Liver
UPENN 056	Liver
UPENN 066	Liver
UPENN 071	Liver
UPENN 060	Liver
UPENN 054	Liver, Peritoneum
UPENN 063	Liver
UPENN 052	Liver, Peritoneum, Omentum
UPENN 055	Liver, Lung
UPENN 051	Liver
UPENN 065	Lung
UPENN 057	Liver
UPENN 068	Liver
UPENN 061	Liver
