# Supplementary material for: Plasma cell‐free DNA markers predict occult metastases in patients with resectable pancreatic ductal adenocarcinoma
Source: Clin Transl Med. 2026 Jan 19;16(1):e70573. doi: 10.1002/ctm2.70573 (PMC12813551; doi:10.1002/ctm2.70573)
Supplement: Supplementary file 4 — Supporting Information [file CTM2-16-e70573-s004.pdf]

Supplemental Table 4 – Sites of first detected occult metastases for patients in naïve resectable cohort with occult metastases (N=25)

ID	Detected by	Site of first detected occult metastases
UPENN 077	Imaging	Liver, cardiophrenic lymph nodes, peritoneal carcinomatosis
UPENN 078	Imaging	Liver
UPENN 079	Imaging	Lung
UPENN 082	Imaging	Liver, peritoneum
UPENN 084	Intraoperative	Liver
UPENN 086	Imaging	Mesenteric lymph nodes
UPENN 088	Imaging	Liver; periportal and portacaval lymph nodes
UPENN 090	Intraoperative	Liver
UPENN 103	Imaging	Liver
UPENN 108	Imaging	Lung
UPENN 112	Imaging	Liver
UPENN 115	Imaging	Lung
UPENN 116	Imaging	Lung; supraclavicular, paratracheal, and precarinal lymph nodes
UPENN 118	Intraoperative	Liver
UPENN 121	Imaging	Peritoneal carcinomatosis
UPENN 122	Imaging	Liver
UPENN 128	Imaging	Liver
UPENN 129	Intraoperative	Liver
UPENN 134	Imaging	Liver
UPENN 139	Intraoperative	Liver
UPENN 140	Intraoperative	Liver
UPENN 142	Imaging	Liver, lung
UPENN 144	Imaging	Liver
UPENN 147	Imaging	Liver, peritoneal carcinomatosis, supraclavicular lymph node
UPENN 149	Imaging	Lung
