# Supplementary material for: Plasma cell‐free DNA markers predict occult metastases in patients with resectable pancreatic ductal adenocarcinoma
Source: Clin Transl Med. 2026 Jan 19;16(1):e70573. doi: 10.1002/ctm2.70573 (PMC12813551; doi:10.1002/ctm2.70573)
Supplement: Supplementary file 5 — Supporting Information [file CTM2-16-e70573-s010.pdf]

Supplemental Table 5 – Patient characteristics for naïve resectable PDAC patients with and without occult metastases

Occult Metastatic Disease	Yes	No	P-value
	25	50	
**Age**			
Median (Min-Max)	71 (50-85)	72 (51-85)	0.5515*
<65, n (%)	4 (16%)	11 (22%)	0.7606**
>65, n (%)	21 (84%)	39 (78%)	
**Sex, n (%)**			
Female	11 (44%)	23 (46%)	>0.9999**
Male	14 (56%)	27 (54%)	
**Race, n (%)**			
African American	5 (20%)	4 (8%)	0.1899**
Asian	0 (0%)	3 (6%)	
Caucasian	20 (80%)	43 (86%)	
**Baseline ECOG Performance Status, n (%)**			
0	3 (12%)	5 (10%)	>0.9999**
1	3 (12%)	6 (12%)	
2	1 (4%)	1 (2%)	
n/a	18 (72%)	38 (76%)	

***Mann-Whitney test, **Fisher's Exact Test**
