# Supplementary material for: Plasma cell‐free DNA markers predict occult metastases in patients with resectable pancreatic ductal adenocarcinoma
Source: Clin Transl Med. 2026 Jan 19;16(1):e70573. doi: 10.1002/ctm2.70573 (PMC12813551; doi:10.1002/ctm2.70573)
Supplement: Supplementary file 7 — Supporting Information [file CTM2-16-e70573-s011.pdf]

Supplemental Table 7 – Primer sequences for cfDNA methylation markers

Gene	Marker	Forward Primer	Reverse Primer	Chr	hg19 location
ATP11A.R	Lung 1	GAGAAGTTAGGAGGAGAGTAGATA	TTTACATTTTAAATTTTATCCC	chr03	113371420
CPNE2	Lung 2	TTTTTTATTTTTTGGGTATTTGT	TAAAAACACTCACATTCCAATAAA	chr16	101493277
DGKD	Lung 3	TTTGTGTGAATAGAAAGATTTTAGTT	AATATAACTCCACCCCAAAATC	chr02	25105451
lungR_meth2	Lung 4	GTAGTTGGGATTTAGAGAAGGTT	AACCCACAACCTAAAATCCTAC	chr05	3594223
lungR_meth3	Lung 5	GGAATTTTGGAGGTTGTAGG	TTATCTTACTAATCATACTACTTCCC	chr05	3592686
lungR_meth4	Lung 6	GGTATAGTGATTAGGGGGTAGTTAT	AAAATAACTAAAACAAACCCTACC	chr05	49031430
lungR-unmeth3	Lung 7	GGAAGTTTTGGTATGATTTTTTT	ACTCTAATATAAACACCTAACAACC	chr01	230139661
lungR-unmeth4	Lung 8	TTTTTTTTGAGATGGGATTT	TAAAAAAATTAAAATTACAATAAACC	chr02	74192569
lungR-unmeth5	Lung 9	GAGAATGAAATAAGATAGGTTTTTTT	ATATAATAAAAATTAAAACCCAACC	chr15	429533
S5-unMe	Lung 10	TAGAGTATTGGTTTGAAGATTTGT	TATCACAACCACACATAAACAAC	chr02	181442317
SOX2-OT	Lung 11	GGGGTTTTAATTTAGGGTTTAG	AATTCACAAATTATTAACAAACACC	chr03	74625730
Pan-ac1	Pancreas 1	GTGGTTTAGTTTTTTGATTTTTTT	AACCCACTACAACAACCTACTATAC	chr16	26190140
Pan-du3	Pancreas 2	TTAGAGGTTAAATTAGGATAAGATTT	TCAACCCAACAAAATTTTCTT	chr06	134499650
Pan-ac3	Pancreas 3	TTGGGATGTTTTTAGTTTTTGT	TCCATAACATTTACCTACAAAAAA	chr10	167192407
Pan-ac9	Pancreas 4	AATTGTTGGGTTTTGTTTTTT	ATCTCACCTAATATTCCCCAAC	chr16	22929881
Pan-du7	Pancreas 5	GAGAAAATGGTTTTAGATTATTGTA	TTAATAAATTAAAATAATATTCACCTC	chr15	25945423
AUTO-1	Pancreas 6	TTTTATTTTAGATTTTAGGAGGAG	AAAAATAACACTACCTAAAAAACC	chr16	68118261
BRF	Pancreas 7	GGTTGATATTATAATTTGTGATAGG	CCAATCCTACTAACTAACCATATC	chr14	105714471
FRY	Pancreas 8	TTTGTAAGGGTTGGTTGTTG	CCTACTTATTTAACCATTTACATTC	chr13	32605843
NOS1	Pancreas 9	TTATAGTGTTTTGGGGGTGG	CAAAACCACTCAAAAACCTTAC	chr12	117798052
cg17952661	Hepatocyte 1	AGTTTTTTTATAATAGTTTTTTGTTAT	ACACTAAAATTTCAAACAAAACTC	chr01	145395716
GPAM	Hepatocyte 2	TTTTTTATTGTTTTAATGTTTTTTAG	TAAACTCAATCCCCTAAATATCTAC	chr10	113943113
IGF2R	Hepatocyte 3	TGGGTGTTGTTATTTTGTTGA	CTACAAAAATACACACCCCAA	chr06	160500566
ITIH4	Hepatocyte 4	ATAGTGAAGATGTTAGTTTGTTTTT	AACACACTTACCTAATAACCAAAC	chr03	52864973
VTN2	Hepatocyte 5	GGTATTTTGAAGAGGTAGGTTT	ACCTAAATACCCCAAACTCAT	chr17	26696304
