# Supplementary figures and images for: Plasma cell‐free DNA markers predict occult metastases in patients with resectable pancreatic ductal adenocarcinoma
Source: Clin Transl Med. 2026 Jan 19;16(1):e70573. doi: 10.1002/ctm2.70573 (PMC12813551; doi:10.1002/ctm2.70573)

Supplemental Figure 1: Study participants

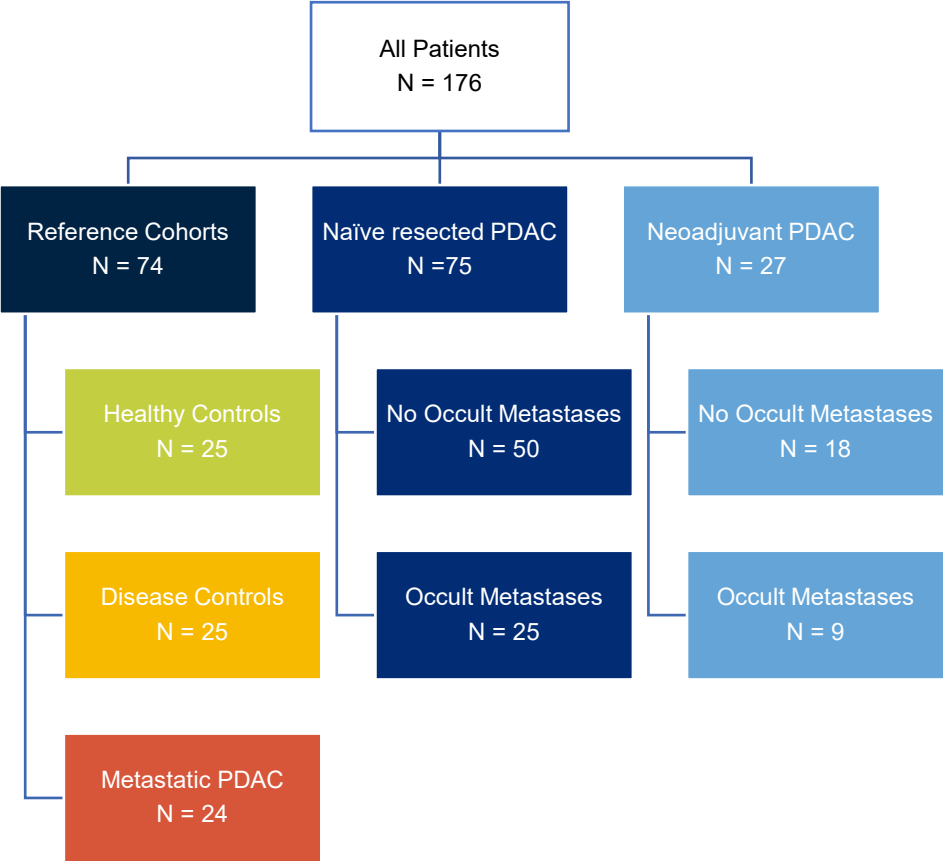

Supplement: Supplementary file 8 — Supporting Information [file CTM2-16-e70573-s001.pdf]
