# Supplementary material for: Plasma cell‐free DNA markers predict occult metastases in patients with resectable pancreatic ductal adenocarcinoma
Source: Clin Transl Med. 2026 Jan 19;16(1):e70573. doi: 10.1002/ctm2.70573 (PMC12813551; doi:10.1002/ctm2.70573)

Supplemental Figure 2 - Tissue specificity and sensitivity of hepatocyte- and lung-derived methylation markers. Methylation status of hepatocyte- and lung-derived markers in genomic DNA across multiple human tissues. Shown in A for hepatocyte-derived markers and C for lung-derived markers is the fraction of molecules that are fully methylated or unmethylated in the indicated tissue/cell type. Each of the colors in A and C represents a locus that is differentially methylated or unmethylated in a specific cell type. Shown in B for hepatocyte- and D for lung-derived markers is the average percentage of molecules with that tissue's specific methylation in genomic DNA across various cell types and tissues.

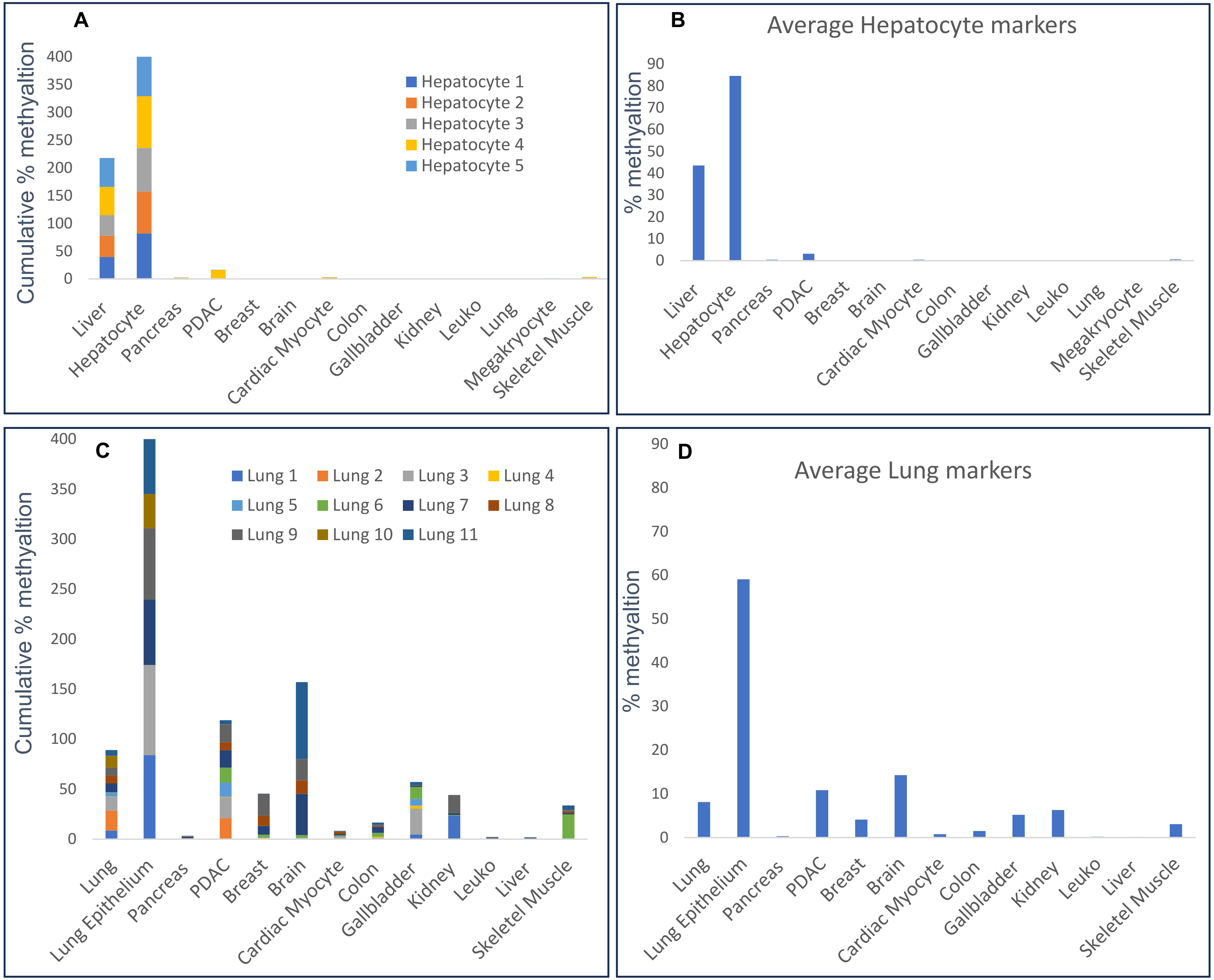

Supplement: Supplementary file 9 — Supporting Information [file CTM2-16-e70573-s012.pdf]
