# Supplementary material for: Plasma cell‐free DNA markers predict occult metastases in patients with resectable pancreatic ductal adenocarcinoma
Source: Clin Transl Med. 2026 Jan 19;16(1):e70573. doi: 10.1002/ctm2.70573 (PMC12813551; doi:10.1002/ctm2.70573)

**Supplemental Figure 3 – Kaplan-Meier analysis for 75 patients with naïve resectable PDAC for overall survival.** Dichotomized at A) with vs without occult metastases, or B) at the median value for each cfDNA methylation biomarker (left) or for pancreas and/or lung copies combined (right).

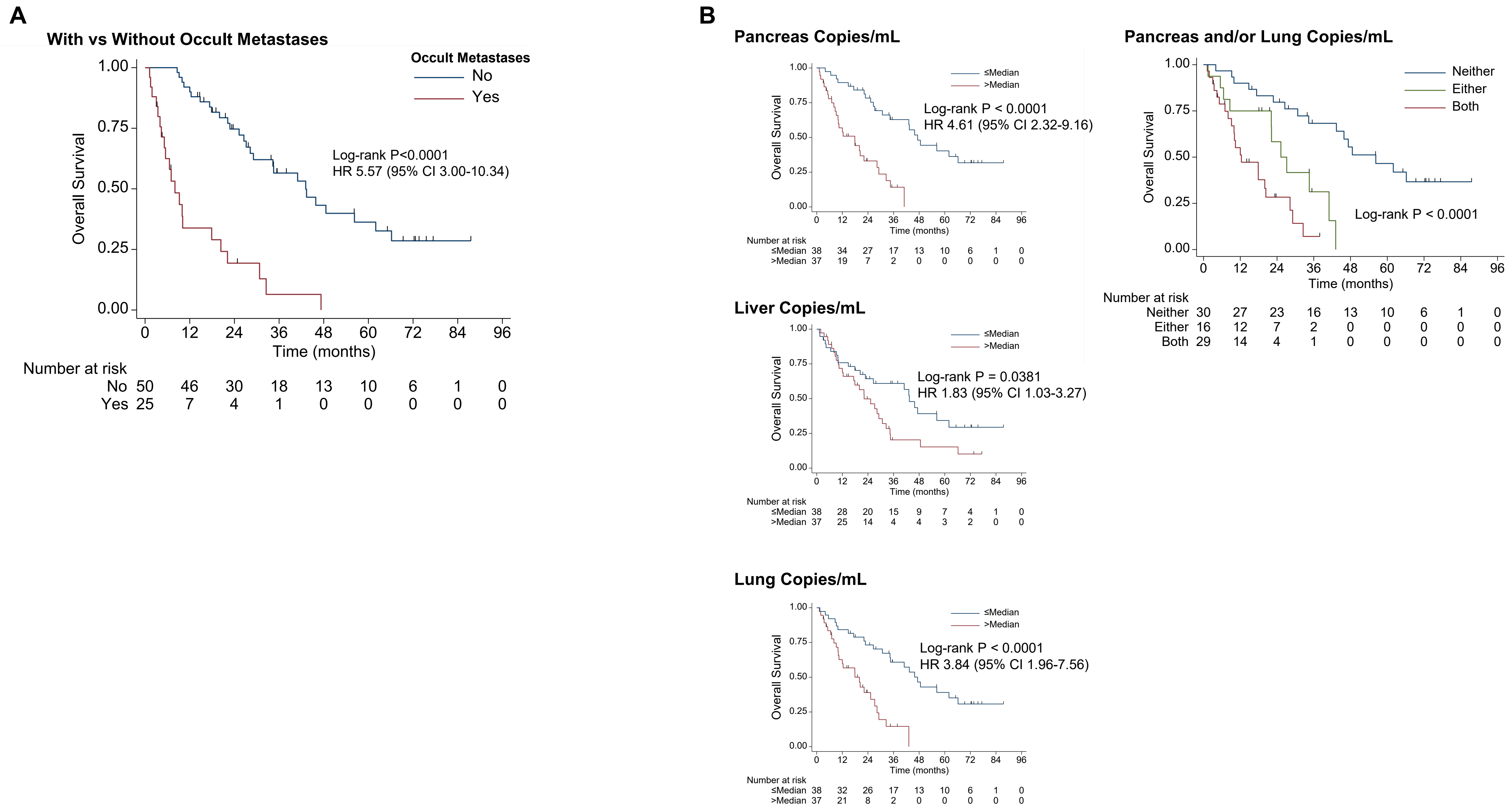

Supplement: Supplementary file 10 — Supporting Information [file CTM2-16-e70573-s013.pdf]
