# Supplementary material for: Plasma cell‐free DNA markers predict occult metastases in patients with resectable pancreatic ductal adenocarcinoma
Source: Clin Transl Med. 2026 Jan 19;16(1):e70573. doi: 10.1002/ctm2.70573 (PMC12813551; doi:10.1002/ctm2.70573)

Supplemental Figure 4 – ROC analysis statistics for cfDNA methylation markers and A) prediction of occult vs no occult metastases among naïve resectable PDAC patients. Shown in B) are the associated Kaplan-Meier analyses dichotomized at the optimal cutoffs for each of the three markers.

A

	Pancreas Copies	Liver Copies	Lung Copies
ROC AUC	0.7356	0.5356	0.6844
ROC 95% CI	0.5998-0.8714	0.3944-0.6768	0.5450-0.8238
Optimal cutoff	> 2.43	> 3.34	> 7.72
Optimal cutoff Likelihood ratio	3.333	1.136	3.25
Optimal cutoff Youden's J index	0.42	0.12	0.36
Sensitivity (%)	60	100	52
Sensitivity 95% CI (%)	40.74-76.60	86.68-100.0	33.50-69.97
Specificity (%)	82	12	84
Specificity 95% CI	69.20-90.23	5.618-23.80	71.49-91.66
Referenced Upper Limit (%)	96.43	2084.5	135.86
Precision	0.625	0.362	0.6
Accuracy	0.747	0.413	0.72

B

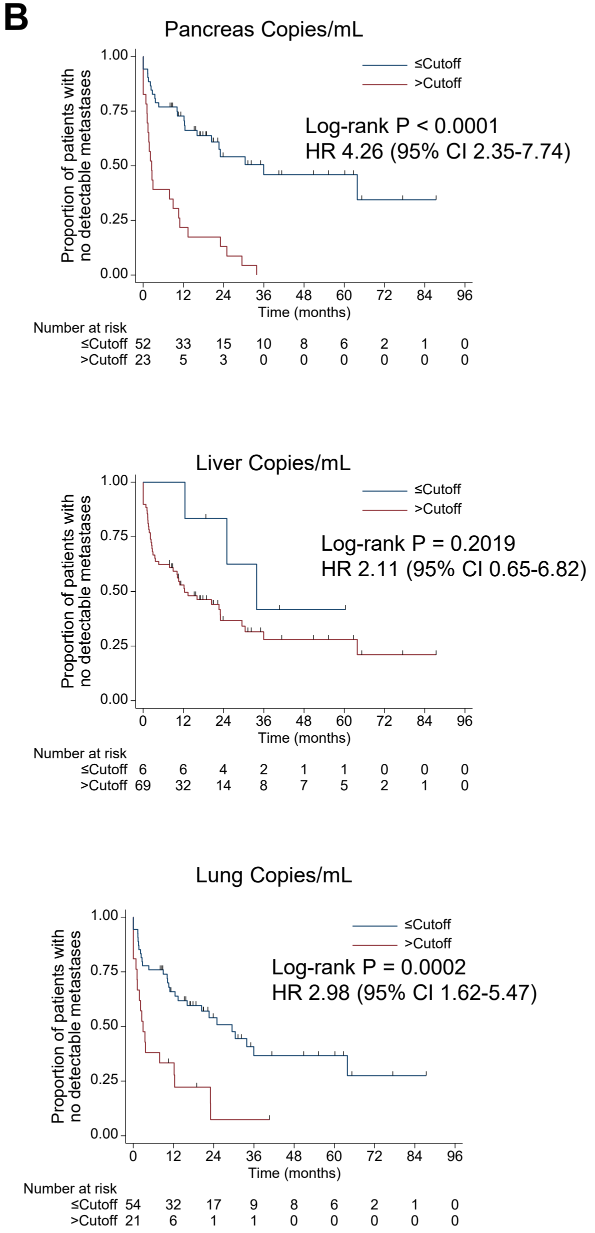

Supplement: Supplementary file 11 — Supporting Information [file CTM2-16-e70573-s014.pdf]
