# Supplementary material for: Plasma cell‐free DNA markers predict occult metastases in patients with resectable pancreatic ductal adenocarcinoma
Source: Clin Transl Med. 2026 Jan 19;16(1):e70573. doi: 10.1002/ctm2.70573 (PMC12813551; doi:10.1002/ctm2.70573)

Supplemental Figure 5 – Kaplan-Meier overall survival analysis for ctKRAS (top), CA19-9 (middle), and tumor volume (bottom).

ctKRAS

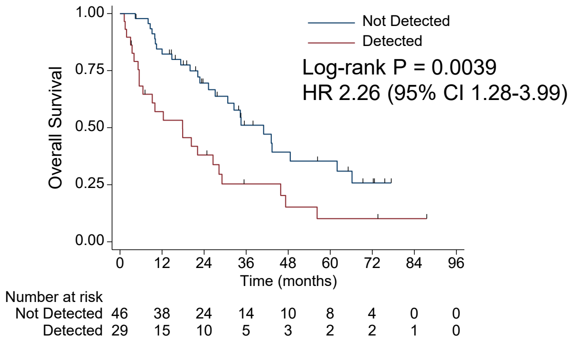

CA19-9

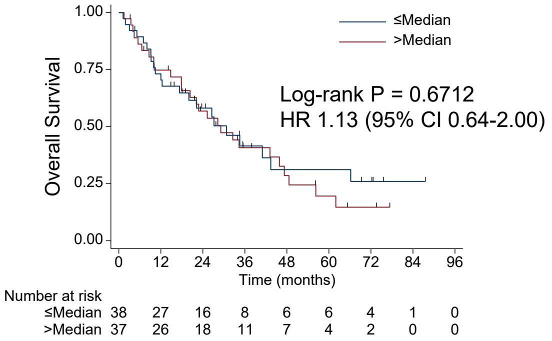

Tumor volume (cm^3)

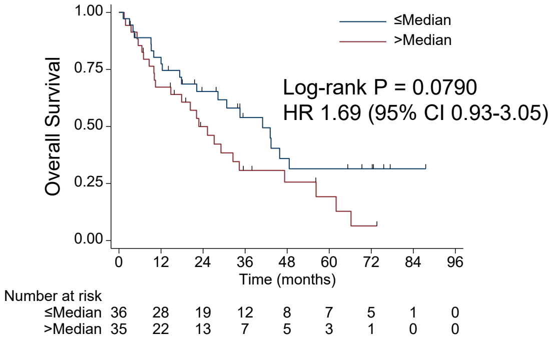

Supplement: Supplementary file 12 — Supporting Information [file CTM2-16-e70573-s009.pdf]
