# Supplementary material for: Plasma cell‐free DNA markers predict occult metastases in patients with resectable pancreatic ductal adenocarcinoma
Source: Clin Transl Med. 2026 Jan 19;16(1):e70573. doi: 10.1002/ctm2.70573 (PMC12813551; doi:10.1002/ctm2.70573)

Supplemental Figure 6 - Biomarker analysis for patients who received neoadjuvant therapy. Shown in **A** are cfDNA biomarkers for blood draws obtained after neoadjuvant therapy but prior to surgery for 27 patients who received neoadjuvant therapy and compared to the 75 patients who received surgery without neoadjuvant therapy ("naïve resectable"). P values generated using Mann-Whitney test. Shown in **B** for the 27 patients who received neoadjuvant therapy are the 3 cfDNA biomarkers comparing those with and without occult metastases. Mann-Whitney P-values for dot plots and ROC curves are at right.

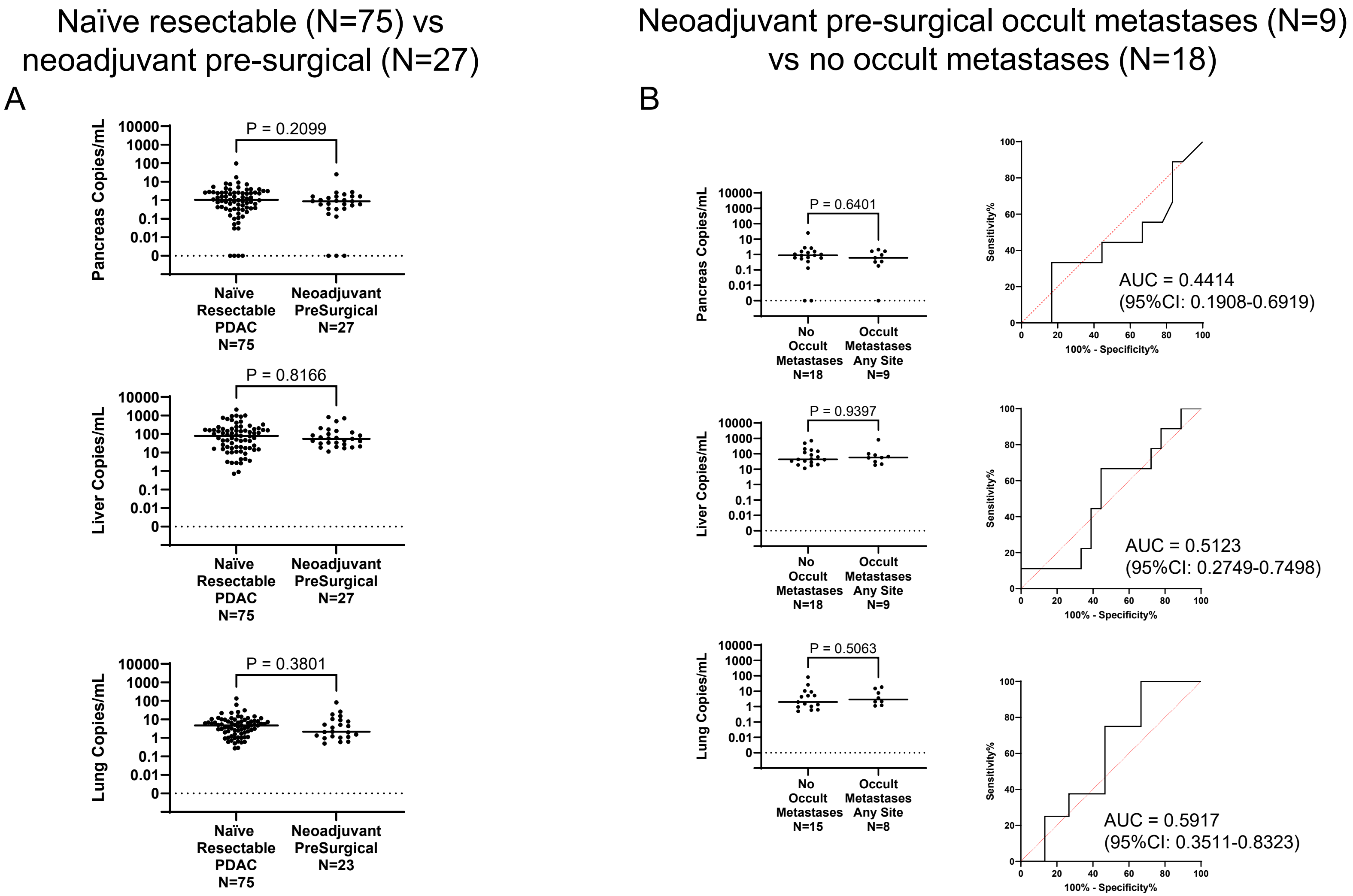

Supplement: Supplementary file 13 — Supporting Information [file CTM2-16-e70573-s005.pdf]
