# Supplementary material for: Plasma cell‐free DNA markers predict occult metastases in patients with resectable pancreatic ductal adenocarcinoma
Source: Clin Transl Med. 2026 Jan 19;16(1):e70573. doi: 10.1002/ctm2.70573 (PMC12813551; doi:10.1002/ctm2.70573)
Supplement: Supplementary file 14 — Supporting Information [file CTM2-16-e70573-s003.docx]

Supplemental Information - Online methods

**Circulating cell-free DNA extraction, quantification, and bisulfite treatment**

Peripheral blood was collected in either K2EDTA vacutainers (Becton Dickinson, New Jersey, USA) or Streck cfDNA tubes (Streck Inc., Nebraska, USA) and processed to plasma within 3 hours or 7 days, respectively, and then banked at -80C. Either cfDNA was first extracted and then shipped, or plasma samples were shipped on dry ice in batches to the Dor lab for cfDNA extraction and analysis (see below). cfDNA was extracted from 2 to 4mL of plasma using either the QIAmp MinElute ccfDNA kit (Qiagen, Inc, Hilden, Germany) or the QIAsymphony liquid handling robot (Qiagen). cfDNA concentration was determined using Qubit double-strand molecular probes kit (Invitrogen) according to the manufacturer’s instructions. DNA derived from all samples was treated with bisulfite using EZ DNA Methylation-Gold (Zymo Research), according to the manufacturer’s instructions, and eluted in 24 μL elution buffer.

**DNA methylation analysis**

A comparative methylome analysis identified five loci that were methylated or unmethylated in hepatocytes and 11 loci that were methylated or unmethylated in lung tissue. Nine pancreatic markers were used as described in Ben Ami et al (1). To validate the specificity of these markers, we amplified the loci from bisulfite-treated genomic DNA derived from a panel of human tissues and sequenced the products, with results shown in **Supplemental Figure 2**. It should be noted that the lung markers are elevated in PDAC tumors, suggesting that the signal observed in the plasma of PDAC patients likely originates from the PDAC tumor and not necessarily from lung tissue. Operators were blinded to cohort and all clinical data associated with samples, including metastatic progression.

**PCR**

To efficiently amplify and sequence multiple targets from bisulfite treated

cfDNA, we used a two-step multiplexed PCR protocol (2). Primer sequences are included in **Supplemental Table 7**. In the first step, up to 10 primer pairs were used in one PCR reaction to amplify regions of interest from bisulfite-treated DNA, independent of methylation status. Primers were 18–30 base pairs (bp) with primer melting temperature ranging from 58C to 62C. To maximize amplification efficiency and minimize primer interference, the primers were designed with additional 25 bp adapters comprising Illumina TruSeq Universal Adapters without index tags. All primers were mixed in the same reaction tube. For each sample, the PCR was prepared using the QIAGEN Multiplex PCR Kit (Qiagen Sciences, Maryland, USA) according to manufacturer’s instructions with 7 μL of bisulfite-treated cfDNA. Reaction conditions for the first round of PCR were: 95C for 15 min, followed by 30 cycles of 95C for 30 s, 57C for 3 min and 72C for 1.5 min, followed by 10min at 68C. In the second PCR step, the products of the first PCR were treated with Exonuclease I (Thermo Fisher Scientific, Massachusetts, USA) for primer removal according to the manufacturer’s instructions. Cleaned PCR products were amplified using one unique TruSeq Universal Adapter primer pair per sample to add a unique index barcode to enable sample pooling for multiplex Illumina sequencing. The PCR was prepared using 2Å~ PCRBIO HS Taq Mix Red Kit (PCR Biosystems, London, England) according to manufacturer’s instructions. Reaction conditions for the second round of PCR were: 95C for 2min, followed by 15 cycles of 95C for 30 s, 59C for 1.5 min, 72C for 30 s, followed by 10 min at 72C. The PCR products were then pooled, run on 3% agarose gels with ethidiumbromide staining, and extracted by Zymo GEL Recovery kit (Zymo Research, California, USA). Operators were blinded to cohort and all clinical data associated with samples, including metastatic progression.

**NGS and analysis of PCR products**

Pooled PCR products were subjected to multiplex NGS using the NextSeq 500/550 v2 Reagent Kit (Illumina). Sequenced reads were separated by barcode and aligned to the target sequence with Bismark. The computational pipeline used to interpret sequence reads as well as a representative set of data were uploaded to GitHub (https:// github. com/ Joshmoss11/btseq (3,4). CpGs were considered methylated if ‘CG’ was read and unmethylated if ‘TG’ was read. Proper bisulfite conversion was assessed by analyzing methylation of non-CpG cytosines. We then determined the fraction of molecules in which all CpG sites were unmethylated. The fraction obtained was multiplied by the concentration of cfDNA measured in each sample, to obtain the concentration of tissue-specific cfDNA from each donor. Given that the mass of a haploid human genome is 3.3pg, the concentration of cfDNA could be converted from units of ng/mL to haploid GE/ml by multiplying by a factor of 303. Our entire dataset of PCR sequencing reactions used in this study is available upon request. Operators were blinded to cohort and all clinical data associated with samples, including metastatic progression.

**ctKRAS**

For ctKRAS detection, separate plasma samples from the methylation analysis described above were used and no bisulfite treatment performed. For the majority of naïve resectable PDAC patients we extracted cfDNA from plasma using the QIAamp Circulating Nucleic Acid Kit (Qiagen) according to manufacturer instructions with two modifications: the proteinase K digestion was extended to 1 hour and final elution was performed twice with 30ul of Buffer AVE (total 60ul). Samples were placed in short-term storage at 4 C. Extracted cfDNA concentration was determined by quantitative PCR for a 115 bp amplicon of human ALU repeat element. Samples were diluted 1:10 with nuclease free water and measured on a standard curve generated by serial dilutions of a commercial standard (Promega G3041). Power SYBR Green PCR Master Mix (Applied Biosystems) was used on QuantStudio 7 Pro Real-Time PCR System (Applied Biosystems) according to manufacturer instructions. Results were analyzed with QuantStudio Design and Analysis Software v2 (Applied Biosystems). Extracted cfDNA was pre-amplified for the *KRAS* G12 locus prior to droplet digital PCR (ddPCR) using Q5 Hot Start Hi-Fidelity Master Mix (NEB) and 0.05uM primers. Maximal cfDNA was added without exceeding 30ng and/or 24ul input. PCR was performed in the Veriti 96 Well Thermal Cycler (Applied Biosystems) and reaction conditions were: 98 C for 3 minutes, 9 cycles of 98 C for 10 seconds, 63 C 3 minutes, and 72 C 30 seconds, followed by 72 C for 2 minutes. Pre-amplified cfDNA was diluted 1:4 with TE buffer and stored at 4 C. ddPCR of pre-amplified material was performed on QX200 (Bio-Rad Laboratories, Inc) platform with *KRAS* G12/G13 Screening Kit (Bio-Rad) using the maximum amount of sample without exceeding 100,000 copies measured and/or 9.9ul. Data was analyzed using QX Manager Software (Bio-Rad). Samples were called positive if the total mutant copies in 20ul ddPCR reaction volume was greater than three standard deviations above the mean for a cohort of healthy control samples. The remaining patients had plasma extracted, and cfDNA quantified, preamplified, and analyzed by ddPCR using previously described methods (5).

**Tumor volume measurement**

For each patient, we identified the unique accession number of the computed tomography (CT) or magnetic resonance imaging (MRI) exam that was obtained immediately prior to the date of surgical tumor resection. All imaging exams were stored in the Sectra Picture Archiving and Communications System (PACS) (Sectra Inc, Connecticut, USA). For the 71 of 75 naïve resectable patients with appropriate imaging available and for all 27 patients who received neoadjuvant therapy, pancreatic tumors were measured in three dimensions by a board-certified radiologist (HS) with over five years of experience post-fellowship, who was blinded to cohort and all other data associated with subjects.The series and image numbers for each measurement were also recorded.

**Statistics**

Kaplan-Meier analysis, Cox Regressions, log-rank test, ROC analyses, and LASSO were performed in Stata/IC 16.1 (StataCorp LLC, Texas, USA., RRID:SCR_012763). Dunn’s Multiple comparisons test, Spearman correlation, ROC analysis, and Mann-Whitney test (all two-sided) were performed in Graphpad Prism 9.5.1 (GraphPad Software, Massachusetts, USA, RRID:SCR_002798). Dunn’s Multiple Comparisons Test was used for comparison of multiple continuous variables with adjustment for multiple testing. Mann-Whitney test and/or Spearman correlation were used for comparison of two continuous variables. Fisher’s Exact Test was used for comparison of two categorical variables. ROC analysis was used for comparison of a continuous variable between two groups, with AUCs and CIs generated in Stata while P-values were generated in GraphPad. Optimal cut-off values were determined by Youden’s J index; if two thresholds had the same index, the threshold with the higher likelihood ratio was selected. All median dichotomizations were performed within the cohort being tested at less than or equal to the median vs greater than the median. Survival statistics were generated using Kaplan-Meier curves to estimate survival function, log-rank test for comparing survival functions between two groups, and Cox regression to determine hazard ratios. For time to metastasis (TTM) analysis, in order to include patients with metastases discovered intraoperatively, time was measured from the day prior to surgery to first detection of metastatic disease or censor. For overall survival analysis, time was measured from day of surgery to date of death or censor. For the LASSO model, the five independent variables were found not to be normally distributed but were log-normal instead. To address this, we log transformed these data (setting zero value specimens to the minimum value for the cohort and variable being analyzed) and standardized by assigning a Z-score, i.e., subtracting the mean and dividing by standard deviation. All LASSO input values were rounded equally to 6 decimals. LASSO was performed with 20-fold cross validation. Median follow-up for TTM for the full naïve and multivariate naïve patients were 1060 (IQR 536-1829) and 1060 (IQR 570-1829) days, respectively. Median follow up for OS for both full naïve and multivariate naïve patients was 1979 (IQR 837-2232) days.

**References for Methods**

1. Ben-Ami R, Wang Q-L, Zhang J, *et al.* Protein biomarkers and alternatively methylated cell-free DNA detect early stage pancreatic cancer. Gut 2024;73:639–648.

2. Neiman D, Gillis D, Piyanzin S, *et al.* Multiplexing DNA methylation markers to detect circulating cell-free DNA derived from human pancreatic **β** cells [Internet]. JCI Insight 2020;5[cited 2024 Nov 7] Available from: https://insight.jci.org/articles/view/136579

3. Moss J, Magenheim J, Neiman D, *et al.* Comprehensive human cell-type methylation atlas reveals origins of circulating cell-free DNA in health and disease. Nat Commun 2018;9:5068.

4. Moss J, Kaplan T, Dor Y. Comprehensive human cell-type methylation atlas reveals origins of circulating cell-free DNA in health and disease [Internet]. GEO Accession Viewer [cited 2024 July 12] Available from: https://www.ncbi.nlm.nih.gov/geo/query/acc.cgi

5. Till JE, McDaniel L, Chang C, *et al.* Circulating KRAS G12D but not G12V is associated with survival in metastatic pancreatic ductal adenocarcinoma. Nat Commun 2024;15:1–12.
