# Supplementary material for: Plasma cell‐free DNA markers predict occult metastases in patients with resectable pancreatic ductal adenocarcinoma
Source: Clin Transl Med. 2026 Jan 19;16(1):e70573. doi: 10.1002/ctm2.70573 (PMC12813551; doi:10.1002/ctm2.70573)
Supplement: Supplementary file 6 — Supporting Information [file CTM2-16-e70573-s006.pdf]

Supplemental Table 6 – Univariate and multivariable analysis for time to metastasis (TTM; top tables) and overall survival (OS; bottom) for cfDNA markers as indicated. Analysis includes independent variables: age (continuous), sex, pathologic tumor stage (pT), pathologic node status (pN), ctKRAS detected vs not detected, log of pre-surgical CA19-9 value (continuous), and log of tumor volume measurements from standard of care pre-surgical imaging (continuous). Among 75 naïve resectable patients, 9 did not have pathologic staging and 4 were missing tumor volume.

Time to Metastasis

Univariate

	HR [95% CI]	P
Pancreas copies/mL	4.23 [2.02-8.86]	0.0001
Lung copies/mL	3.00 [1.46-6.16]	0.0029
Pancreas/Lung copies/mL (Neither, Either, Both)	2.33 [1.52-3.58]	0.0001
Age	0.98 [0.94-1.02]	0.2509
Sex	0.93 [0.48-1.81]	0.8392
pT (1/2 vs 3/4)	1.19 [0.62-2.30]	0.6051
pN (0 vs 1/2)	2.54 [0.98-6.59]	0.0549
ctKRAS	3.06 [1.58-5.90]	0.0009
log CA19-9	1.24 [0.79-1.94]	0.3461
log Tumor Volume (cm^3)	1.74 [0.84-3.60]	0.1363

Multivariable

	HR [95% CI]	P
Pancreas copies/mL	3.24 [1.42-7.42]	0.0054
Age	1.01 [0.96-1.05]	0.7497
Sex	1.35 [0.65-2.81]	0.4141
pT (1/2 vs 3/4)	0.88 [0.43-1.79]	0.7215
pN (0 vs 1/2)	3.93 [1.29-11.92]	0.0157
ctKRAS	3.30 [1.57-6.93]	0.0016
log CA19-9	0.86 [0.56-1.34]	0.5126
log Tumor Vol. (cm^3)	1.60 [0.69-3.70]	0.2732

	HR [95% CI]	P
Lung copies/mL	2.21 [0.98-4.96]	0.0546
Age	0.99 [0.95-1.03]	0.6364
Sex	1.08 [0.54-2.19]	0.8221
pT (1/2 vs 3/4)	0.86 [0.42-1.76]	0.6744
pN (0 vs 1/2)	3.18 [1.01-9.99]	0.0480
ctKRAS	3.90 [1.85-8.23]	0.0004
log CA19-9	0.95 [0.61-1.49]	0.8347
log Tumor Vol. (cm^3)	1.22 [0.54-2.77]	0.6392

	HR [95% CI]	P
Pancreas/Lung copies/mL (Neither, Either, Both)	1.85 [1.17-2.94]	0.0089
Age	1.00 [0.95-1.04]	0.8918
Sex	1.23 [0.60-2.52]	0.5705
pT (1/2 vs 3/4)	0.83 [0.40-1.71]	0.6169
pN (0 vs 1/2)	3.16 [1.04-9.64]	0.0433
ctKRAS	3.38 [1.61-7.10]	0.0013
log CA19-9	0.92 [0.59-1.42]	0.6972
log Tumor Volume (cm^3)	1.40 [0.62-3.17]	0.4171

Overall Survival

Univariate

	HR [95% CI]	P
Pancreas copies/mL	4.08 [1.87-8.91]	0.0004
Lung copies/mL	4.36 [1.94-9.82]	0.0004
Pancreas/Lung copies/mL (Neither, Either, Both)	2.59 [1.63-4.10]	0.0001
Age	1.00 [0.96-1.04]	0.8387
Sex	0.95 [0.49-1.84]	0.8801
pT (1/2 vs 3/4)	1.75 [0.89-3.43]	0.1019
pN (0 vs 1/2)	3.43 [1.20-9.79]	0.0212
ctKRAS	2.05 [1.05-3.98]	0.0345
log CA19-9	1.03 [0.66-1.61]	0.8808
log Tumor Volume (cm^3)	2.47 [1.12-5.43]	0.0250

Multivariable

	HR [95% CI]	P
Pancreas copies/mL	4.30 [1.83-10.06]	0.0008
Age	1.06 [1.01-1.11]	0.0292
Sex	1.45 [0.70-3.00]	0.3202
pT (1/2 vs 3/4)	1.52 [0.73-3.14]	0.2643
pN (0 vs 1/2)	8.31 [2.19-31.60]	0.0019
ctKRAS	2.99 [1.33-6.70]	0.0079
log CA19-9	0.51 [0.32-0.81]	0.0045
log Tumor Vol. (cm^3)	3.82 [1.38-10.58]	0.0098

	HR [95% CI]	P
Lung copies/mL	2.93 [1.22-7.02]	0.0158
Age	1.03 [0.98-1.07]	0.2474
Sex	1.09 [0.53-2.21]	0.8207
pT (1/2 vs 3/4)	1.70 [0.83-3.52]	0.1488
pN (0 vs 1/2)	5.65 [1.41-22.68]	0.0145
ctKRAS	3.56 [1.59-7.98]	0.0021
log CA19-9	0.60 [0.37-0.95]	0.0313
log Tumor Vol. (cm^3)	2.19 [0.84-5.70]	0.1098

	HR [95% CI]	P
Pancreas/Lung copies/mL (Neither, Either, Both)	2.35 [1.40-3.94]	0.0012
Age	1.04 [0.99-1.09]	0.0892
Sex	1.19 [0.58-2.44]	0.6275
pT (1/2 vs 3/4)	1.63 [0.79-3.36]	0.1875
pN (0 vs 1/2)	5.82 [1.53-22.24]	0.0100
ctKRAS	2.96 [1.33-6.61]	0.0080
log CA19-9	0.54 [0.34-0.86]	0.0094
log Tumor Volume (cm^3)	2.83 [1.09-7.36]	0.0328
